# Waterlogging Tolerance at Germination in Field Pea: Variability, Genetic Control, and Indirect Selection

**DOI:** 10.3389/fpls.2019.00953

**Published:** 2019-07-30

**Authors:** Md Shahin Uz Zaman, Al Imran Malik, Parwinder Kaur, Federico Martin Ribalta, William Erskine

**Affiliations:** ^1^Centre for Plant Genetics and Breeding, UWA School of Agriculture and Environment, The University of Western Australia, Crawley, WA, Australia; ^2^The UWA Institute of Agriculture, The University of Western Australia, Crawley, WA, Australia; ^3^Pulses Research Centre, Bangladesh Agricultural Research Institute, Ishwardi, Bangladesh

**Keywords:** germination, waterlogging tolerance, indirect selection, secondary traits, *Pisum* sp.

## Abstract

In the Eastern Gangetic Plain of South Asia field pea (*Pisum sativum* L.) is often grown as a relay crop where soil waterlogging (WL) causes germination failure. To assess if selection for WL tolerance is feasible, we studied the response to WL stress at germination stage in a recombinant inbred line (RIL) population from a bi-parental cross between WL-contrasting parents and in a diversity panel to identify extreme phenotypes, understand the genetics of WL tolerance and find traits for possible use in indirect selection. The RIL population and the diversity panel were screened to test the ability of germination under both waterlogged and drained soils. A total of 50, most WL tolerant and sensitive, genotypes from each of both the RIL and the diversity panel were further evaluated to assay testa integrity/leakage in CaSO_4_ solution. Morphological characterization of both populations was undertaken. A wide range of variation in the ability to germination in waterlogged soil was observed in the RIL population (6–93%) and the diversity panel (5–100%) with a high broad-sense heritability (*H*^2^ > 85%). The variation was continuously distributed indicating polygenic control. Most genotypes with a dark colored testa (90%) were WL tolerant, whereas those with a light colored testa were all WL sensitive in both the RIL population and diversity panel. Testa integrity, measured by electrical conductivity of the leakage solute, was strongly associated with WL tolerance in the RIL population (*r*_G_ = −1.00) and the diversity panel (*r*_G_ = −0.90). Therefore, testa integrity can be effectively used in indirect selection for WL tolerance. Response to selection for WL tolerance at germination is confidently predicted enabling the adaptation of the ancient model pea to extreme precipitation events at germination.

## Introduction

Peas (*Pisum sativum* L.) are an important pulse crop, ranks second in global production after beans among the pulse crops (Food and Agriculture Organization [FAO], 2017). Pea seeds are rich in protein, slowly digestible starch, soluble sugars, fiber, minerals, and vitamins (Dahl et al., 2012). It has an economic and agronomic importance in cropping systems (Yang et al., 2018). The crop is also an important component of agroecological cropping systems in diverse regions of the world. In South Asia, there is a history of relay-sowing of pea into standing rice on waterlogged soil (Ali and Sarker, 2013). Waterlogging (WL) can cause germination failure (Crawford, 1977) and lead to reduced plant population in pea (Zaman et al., 2018).

Global climate change causes WL events to be more frequent, severe, and unpredictable (Intergovernmental Panel on Climate Change [IPCC], 2014). Climate change predictions for South Asia suggest alterations in the intensity of rainfall events, an increase in inter-annual precipitation variability (Sivakumar and Stefanski, 2010), and delayed monsoon rains (Li et al., 2017). This constitutes a major threat to regional crop production. Pea is very prone to WL, even more than other grain legumes (Solaiman et al., 2007; Pampana et al., 2016). In recent years, unseasonal rain during sowing exposed the pea crop to WL stress (Zaman et al., 2018). Therefore, it is crucial to develop stress-resistant peas and to improve agricultural practices to cope with WL stress.

Developing pea genotypes tolerant to WL might be an effective strategy to mitigate WL stress. Variation in WL tolerance at germination among three pea genotypes was demonstrated by Zaman et al. (2018) indicative of valuable diversity within the species. WL tolerance at germination has also been identified in lentil (*Lens culinaris* Medik. ssp. *culinaris*) (Wiraguna et al., 2017), pigeonpea (*Cajanus cajan* (L.) Millsp.) (Sultana et al., 2013), soybean (*Glycine max* (L.) Merr.) (Hou and Thseng, 1991), wheat (*Triticum aestivum* L.) (Ueno and Takahashi, 1997), maize (*Zea mays* L.) (Zaidi et al., 2012), and barley (*Hordeum vulgare* L.) (Takeda and Fukuyama, 1986). However, the long history of focused breeding on high yield and food quality has led to a loss of genetic diversity and stress resistance. Therefore, breeders have to undertake more efficient methods of selection and take advantage of the large genetic diversity present in pea genepool. Recently, Simple Sequence Repeat marker panels have been developed that could be useful for identifying markers linked to WL tolerance and marker-assisted selection (Burstin et al., 2015), but no markers linked to WL tolerance have been identified yet. The value of morpho-physiological traits as indirect selection criteria for WL tolerance is also worthy of evaluation. Several traits are associated with WL tolerance at germination. Small seeds in soybean showed a higher germination rate than large seeds when exposed to WL (Sayama et al., 2009). Testa (seed coat) color is sometimes associated with WL tolerance (Hou and Thseng, 1991; Ueno and Takahashi, 1997; Zhang et al., 2008). Several studies on the role of the testa in preventing cellular damage during imbibition showed that seeds with cracked testa and seeds without testa had rapid imbibition and higher solute leakage than those with intact testa and no cracks [Larson, 1968, pea; Powell and Matthews, 1978, pea; Duke and Kakefuda, 1981, soybean, navy bean (*Phaseolus vulgaris L*.), pea, and peanut (*Arachis hypogaea L*.); Duke et al., 1983, soybean]. Furthermore, a short period (i.e., 24 h) of seed submergence showed rapid imbibition leading to solute leakage, and was associated with low seedling vigor (Perry and Harrison, 1970, pea; Yaklich et al., 1979, soybean; and Kantar et al., 1996, faba bean). Testa integrity appears to be a key trait for WL tolerance at germination.

Here, to assess if selection for WL tolerance is feasible in peas, we studied the response to WL stress at germination stage in a recombinant inbred line (RIL) population from a bi-parental cross between WL-contrasting parents and a diversity panel to: (i) identify extreme phenotypes for WL tolerance, (ii) understand the genetic basis of WL tolerance, and (iii) find traits for possible use in indirect selection for WL tolerance.

## Materials and Methods

### Plant Materials

A RIL population and a diversity panel of pea germplasm were used in this study.

The RIL population (108 lines) originated from a bi-parental cross between WL-tolerant genotype Kaspa and sensitive BM-3 (Zaman et al., 2018). Hybridization was done at the University of Western Australia (UWA) in 2015. The F_1_ generation was allowed to self-pollinate and 250 F_2_ seeds were produced in the glasshouse at an average temperature of 25^∘^C in 2016. Generation advancement from F_2_ to F_6_ was undertaken by a rapid generation system using single seed descent from May 2016 to June 2017. The rapid generation acceleration involves growing the plants under conditions optimized to induce rapid flowering, tagging the flowers at anthesis, then removing pods from the plant prior to treatment to induce precocious germination. The second generation plants are then returned to soil and the process repeated until the desired generations are achieved. Seeds from the last generation are then left to mature on the plant. Plants were grown under far red-enriched LED light (AP67 spectrum) from B series Valoya lights (Helsinki, Finland), with the temperature set at 24/20^∘^C and a photoperiod of 20 h (Croser et al., 2016; Ribalta et al., 2017). Seeds were sown in 0.4 L plastic pots filled with steam pasteurized potting mix (UWA Plant Bio Mix – Richgro Garden Products Australia Pty Ltd.). Plants were watered daily and fertilized weekly with a water soluble N–P–K fertilizer (19–8.3–15.8) with micronutrients (Poly-feed, Greenhouse Grade, Haifa Chemicals Ltd.) at a rate of 0.3 g per pot.

The diversity panel of 110 genotypes comprised five Australian varieties and germplasm accessions from the Australian Temperate Field Crops Collection (ATFCC), Department of Primary Industries, Victoria. The panel included the WL contrasting genotypes – Kaspa and BM-3. The germplasm represents global pea diversity and originates from the geographic regions of South Asia (21 genotypes), former USSR (18), Northern Europe (18), Mediterranean (17), North America (12), Australia (9), South America (8), and Africa (7).

### Methods

Three types of experiments and within each a RIL population and diversity panel trial were conducted.

#### Experiment 1: Studies on Waterlogging Tolerance

##### RIL population

The experiment to assay WL tolerance was conducted in a glasshouse of the Plant Growth Facility at UWA as in Zaman et al. (2018) using the 108 RIL population and the parents – tolerant Kaspa and sensitive BM-3. The experimental design was split-plot with three replicate blocks. Main plots were WL treatments [two levels: drained control and 8 d (days) WL] while the genotypes (108 RILs and two parents) were in sub-plots. The experimental unit was plastic pot-free draining with a sealed base. Free-draining pots contained gravel at the bottom and 3.5 kg sand and soil mixed (1:1) [pH 6.7 and electrical conductivity (EC) 0.46 dS m^–1^ at 1:5 *w*/*v* soil/water] at the top. Soil was collected from Mukinbudin (30^∘^78′ S, 118^∘^31′ E), Western Australia (Kotula et al., 2015). Each free-draining pot (19-cm height × 21-cm diameter) was placed in a sealed base pot (24-cm height × 26-cm diameter). Platinum (Pt) electrodes were inserted in the substrate at a depth of 100 mm in 10 pots for redox measurement (Patrick et al., 1996). For the waterlogged treatment, DI water was added to sealed pots so that free-draining pots could be waterlogged from the bottom to maintain a water table at 10 mm below the soil surface. Pots were waterlogged for 4 d prior to sowing to ensure hypoxia at sowing. Water was added to sealed base pots daily as required to maintain the water table. For drained control treatments, there was no water in the sealed base pots, but the soil moisture in free-draining pots was maintained at ∼80% of field capacity. Seeds were treated with tetramethylthiuram disulfide (Thiram) at the rate of 3 g/kg seeds just before sowing. Twenty seeds of each genotype were sown in a free-draining pot by dibbling at 5 mm soil depth. The seed rate for WL screening followed the WL-screening protocol of Zaman et al. (2018). All pots were covered for 3 d after sowing to ensure darkness for germination. Waterlogged pots were drained after 8 d of WL treatment. Drained control pots were weighed every day and watered to ∼80% field capacity. Within replicates, pots were moved every 5 d to minimize the effects of varying conditions in the glasshouse. The experiment was conducted at 25^∘^C temperature and was terminated 23 d after sowing, when there was no sign of further emergence.

##### Diversity panel

The experiment to assay WL tolerance was conducted on 110 genotypes of the diversity panel including the WL controls – WL-tolerant Kaspa and WL-sensitive BM-3 at germination under similar growth conditions and management practices as described above for the RIL population. The experimental design was split-plot in three replicate blocks with WL treatments (as above) as main plots and genotypes as sub-plots.

Seed emergence was recorded daily during WL and during the recovery period (draining of pots after 8 d WL); and expressed as a percentage of the total number of seeds sown. The emergence was assessed till 23 d, the end day of experiment. Seeds with an epicotyl longer than 5 mm were considered as germinated (i.e., emerged). The redox potential of the soil was recorded daily from 10 pots with a Pt electrode and silver/silver chloride reference electrode attached to a millivolt-meter following the procedure described by Patrick et al. (1996).

#### Experiment 2: Agro-Morphological Traits and WL Tolerance

##### RIL population

The RIL population (108 lines) and two parents were screened for agro-morphological traits in the UWA glasshouse from May to September 2017 in a randomized complete block design with two replications. The experimental unit was plastic pot (diameter 26 cm and height 23 cm). Each pot was filled with gravel at the bottom with 4.0 kg of potting mix (composition described above) on top. Five seeds of each genotype were sown in each pot. After 3 weeks, plants were thinned to two plants per pot. Four weeks after sowing, a water soluble N–P–K fertilizer (19–8.3–15.8) with micronutrients (Poly-feed, Greenhouse Grade, Haifa Chemicals Ltd.) at a rate of 0.3 g per pot were applied and this concentration was doubled after 6 weeks. The fertilizer was applied weekly until the end of grain filling. Insecticide Spinetoram (DOW Agrosciences Australia Limited) was applied as required to control fungal gnat larvae (*Orfelia* and *Bradysia* sp.). Pots were watered to ensure the plants had access to adequate moisture. Watering was stopped to individual pots when pod color turned to light yellow. The average temperature of the glasshouse was 18^∘^C from May to September 2017.

##### Diversity panel

The diversity panel (110 genotypes including two controls – WL-tolerant Kaspa and WL-sensitive BM-3) was screened for agro-morphological traits in the UWA glasshouse in a randomized complete block design with two replications. Seed sowing and other management practices were the same as for the RIL population above. The experiment was conducted in the UWA glasshouse with an average temperature of 22^∘^C from September to December 2016.

Stem base width and plant height of five plants were measured 3 weeks after sowing using digital Vernier caliper (Kincrome, Australia) and 30-cm plastic scale (Promotion Products, Australia), respectively. Flower color and leaf axil pigmentation were noted at flowering using UPOV pea descriptors (International Union for the Protection of New Varieties of Plants [UPOV], 2009) were used with 1–3 (1 = white, 2 = pink, and 3 = purple) and 1–2 (1 = absent and 2 = present as single ring) scoring scales, respectively. Time to 50% flowering (d) was recorded for individual plants. Testa color and seed weight were recorded after drying for 3 months at room temperature following harvest. Testa color was scored with a 1–9 (1 = light yellow, 2 = yellow pink, 3 = waxy, 4 = yellow–green, 5 = gray–green, 6 = dark green, 7 = light brown, 8 = brown, and 9 = black) scoring scale (Pavelkova et al., 1986). The color of flower, leaf axil, and testa was observed and confirmed by horticultural color chart (Wilson, 1942).

#### Experiment 3: Testa Leakage and WL Tolerance

##### RIL population

To assay for testa integrity/leakage under WL conditions 50 genotypes with contrasting responses (i.e., 25 tolerant and 25 sensitive RIL lines) to WL treatment were selected from the 108 RIL population. Testa color was categorized into two groups based on scoring scale of Pavelkova et al. (1986), where 1–6 score for light and 7–8 for dark colored testa. In the WL-tolerant group all genotypes had dark testa, but the sensitive group comprised 3 dark and 22 light testa genotypes. The testa of tolerant parent (Kaspa) was dark in color, whereas the sensitive parent BM-3 had a light colored testa. The 50 genotypes were subjected to a submergence treatment with eight replications in a completely randomized design. An individual seed representing a replicate of each genotype was submerged in a 50 ml centrifuge tube (SARSTEDT, Germany) containing 40 ml of 0.5 mM CaSO_4_ solution and incubated in a germination cabinet at 25^∘^C temperature with 12:12 light–dark cycle for 6 d.

##### Diversity panel

A total of 50 genotypes with contrasting responses (i.e., 25 tolerant and 25 sensitive) to WL were selected from the 110-genotype diversity panel. The tolerant 25 genotypes comprised 20 dark and 5 light colored testa, whereas sensitive 25 genotypes comprised of 23 light and 2 dark testa. Experimental design and growth conditions were similar to that of the RIL population.

Electrical conductivity of submergence solution was measured after 6 d of treatment with an AQUA-PH v1.0 conductivity meter (TPS, Brisbane, QLD, Australia). Seeds were germinated in CaSO_4_ solution so the germination was counted at the end of the experiment on day 6. Seeds with a radicles longer than 3 mm were considered as germinated. Germination was reported in percent based on the number of seeds germinated from eight seeds of each genotypes.

### Statistical Analysis

Data were analyzed using GenStat 16th edition for Windows statistical software (VSN International, United Kingdom). Analyses of variance (ANOVAs) were undertaken to determine the effects of the different treatments, and least significant differences (l.s.d) at *P* > 0.05 calculated for significant differences between treatments, genotypes, and interaction means. A one-way ANOVA was also conducted by region of origin. Spearman’s rank correlation coefficient was calculated by STAR statistical software, version 2.0.1 2014 (Biometrics and Breeding Informatics, PBGB Division, International Rice Research Institute, Los Baños, Philippines). Chi-square tests for goodness-of-fit was conducted to measure the inheritance of testa color.

### Response to Selection

The broad-sense heritability was estimated by: $\sigma_{g}^{2}=\left(\sigma_{g}^{2}\right)/\left(\sigma_{g}^{2}+\sigma_{e}^{2}\right)$, where $\sigma_{g}^{2}$ and $\sigma_{e}^{2}$ are the estimated genotypic and error variances, respectively (Nyquist and Baker, 1991). The estimated genotypic and error variances were calculated as: σ^2^_g_ = (MS_g_ – MS_e_)/*r* while $\sigma_{e}^{2}$ = MS_e_/*r*, where MS_g_ is the mean square of the population, MS_e_ is the residual error, and *r* is the number of replicates.

Genetic correlations between traits were computed as: *r*_G12_ = *r*_P12_/√($H_{1}^{2}$ × $H_{2}^{2}$) (Cooper et al., 1996) where *r*_G12_, *r*_P12_, $H_{1}^{2}$, and $H_{2}^{2}$ are the genotypic correlation between traits 1 and 2, phenotypic correlation between the same pair of traits, and heritability of traits 1 and 2, respectively.

The efficiency of indirect selection was estimated as (Cooper et al., 1996; Kumar et al., 2008): CR_G_/DR_G_ = *r*_G_√($H_{s}^{2}$/$H_{g}^{2}$); where CR_G_ is the correlated response to indirect selection for germination based on secondary traits, DR_G_ indicates direct response to selection for germination, *r*_G_ is the genotypic correlation, $H_{s}^{2}$ and $H_{g}^{2}$ represent heritability for the secondary trait and germination, respectively, under waterlogged stress.

## Results

### Redox Measurements

In drained control soil the redox potential in the RIL population was 585 ± 5 mV throughout the experimental period. By contrast, the redox potential in waterlogged pots was 318 ± 6 mV throughout the WL period and this increased on draining the pots to 565 ± 13 by 23 d. In the experiment with the diversity panel the redox potential in drained and waterlogged pots followed the same trend as for RIL population experiment.

### Variation of Waterlogging Tolerance

**FIGURE 1:**
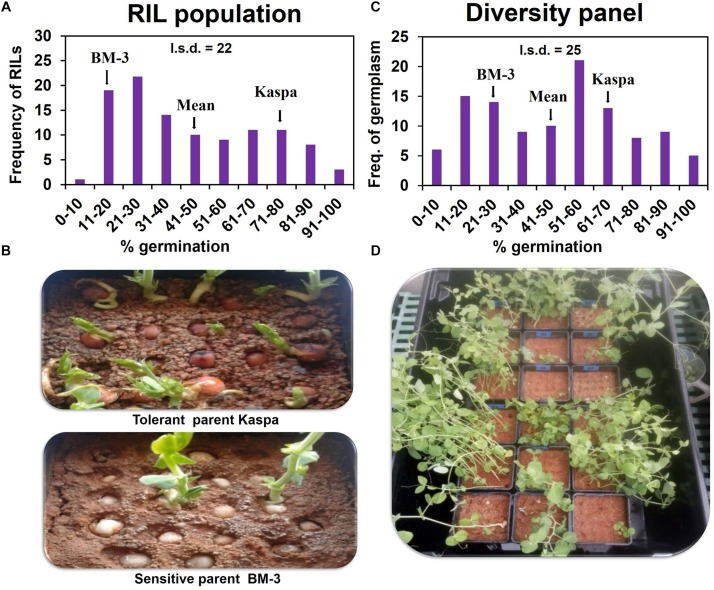
Variation of germination/WL tolerance on 8 d waterlogged soil in RIL population **(A,B)** and diversity panel **(C,D)**. Emergence started from 3 d after sowing and was completed by 23 d. Germination was recorded daily for each seed in both the waterlogged and recovery periods. Seeds with an epicotyl longer than 5 mm were considered as germinated. The l.s.d. is at *P* = 0.05 and *n* = 324 and 330 for RIL population and diversity panel, respectively.

In the RIL population, all the genotypes including parents showed close to 100% germination in drained soil. However, in waterlogged soil the RIL parents showed contrasting responses in germination (measured as emergence) − tolerant Kaspa 73% and sensitive BM-3 20% (LSD*_P_*_=__0.05_ = 22). The population of 108 RIL lines exhibited segregation from 6 to 93% germination (Figures 1A,B). The mean germination of the RIL population was 41%, mid-way between the parents. Significant transgressive segregation was not recorded in either direction. A high broad-sense heritability of *H*^2^ = 89% was found for germination under waterlogged conditions for this RIL population.

In the experiment with the diversity panel, in drained soil all the genotypes (controls) showed close to 100% germination. However, in waterlogged soil a wide range in germination was observed from 5 to 100% exhibiting a continuous distribution (Figures 1C,D). The mean for germination in the diversity panel was 48%, mid-way between tolerant control Kaspa (68%), and sensitive control BM-3 (22%) (LSD*_P_*_=__0.05_ = 25). Five genotypes significantly (*P* < 0.05) exceeded the tolerant parent Kaspa in germination under WL, but no genotype was significantly less tolerant than the sensitive BM-3 control. In the diversity panel the broad-sense heritability for germination in waterlogged soil was high at *H*^2^ = 87%.

**FIGURE 2:**
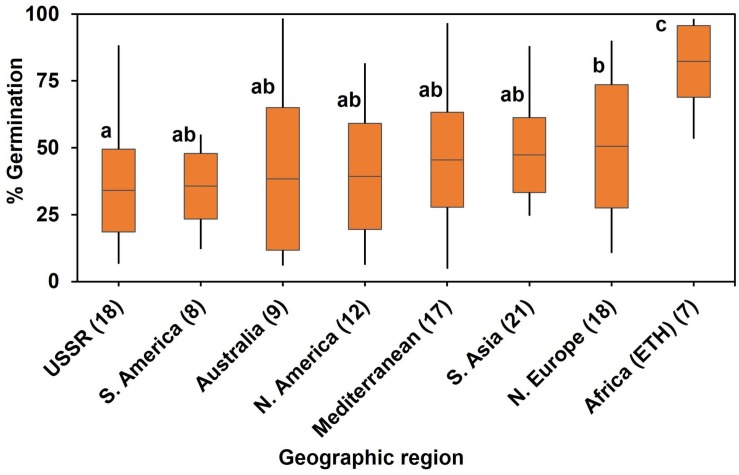
Association of percent germination/WL tolerance with geographic region of origin after 8 d of soil WL. Box plot represents mean germination (mid-point of box plot), standard error (box plot length), together with minimum and maximum values (whisker bars). Multiple comparison was (l.s.d., *P* = 0.05) based on one-way ANOVA (*P* < 0.001) by geographic region of origin. Means followed by different letters are significantly different at *P* = 0.05. The number in brackets denotes the number of genotypes in a region.

In the diversity panel, a one-way ANOVA by region of origin showed that geographic region accounted for significant (*P* < 0.001) variation in WL tolerance at germination (Figure 2). Genotypes from Africa (i.e., Ethiopia in the current study) showed highest germination (80%) on average when exposed to soil waterlogged. The poorest performance under waterlogged conditions was from genotypes from the former USSR.

### Morphological Traits and Waterlogging Tolerance

**FIGURE 3:**
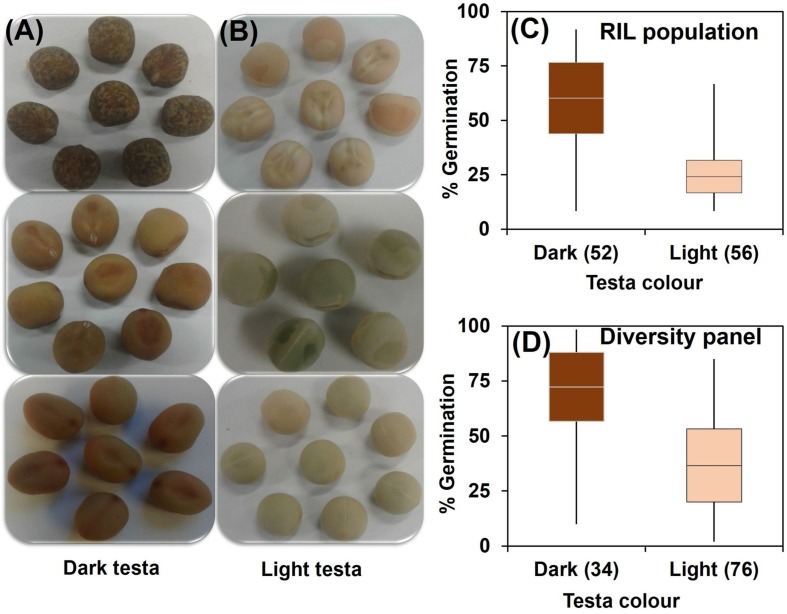
Categorization of testa color into **(A)** dark and **(B)** light; and distribution of genotypes based on testa color and germination from waterlogged soil for **(C)** RIL population and **(D)** diversity panel. Box plot represents mean germination (mid-point of box plot), standard error (box plot length), together with minimum and maximum values (whisker bars). The number in parentheses denotes the number of genotypes per group.

Correlation coefficients showed pair-wise associations between WL tolerance and morphological traits as well as among the morphological traits (Table 1). In the RIL population the strongest positive correlations with WL tolerance were found for the three traits – flower color (*r* = 0.62), leaf axil pigmentation (*r* = 0.66), and testa color (*r* = 0.59) (Table 1A). Furthermore, a detailed analysis of testa color showed two distinct parental groups: dark like WL-tolerant Kaspa (Figure 3A) and light testa like WL-sensitive BM-3 (Figure 3B). Overall, 52 of the 108 genotypes had dark colored and the rest 56 was light-colored testa that segregated in 1:1 ratio (χ^2^ = 0.15, *P <* 0.001), indicating single gene controlling the trait (Figure 3C). The average germination of dark testa RIL genotypes was 58%, which was significantly (*P* < 0.001) higher than the mean for genotypes with light-colored testa (26%). The range of percent germination was from 8 to 92% for dark and 8 to 65% for light testa genotypes.

**TABLE 1:** Spearman’s rank phenotypic correlation coefficients (*r*) between morphological traits for (A) RIL population (*n* = 108) and (B) diversity panel (*n* = 110 genotypes).

**Traits**	**Waterlogged germination**	**Flower color (FC)**	**Leaf axil pigment (LAP)**	**Testa color (TC)**	**Seed weight (SW)**	**Stem base width (SBW)**	**Plant height (PH)**
**(A) RIL population**							
FC	**0.62^∗∗∗^**	**−**					
LAP	**0.66^∗∗∗^**	**0.95^∗∗∗^**	**−**				
TC	**0.59^∗∗∗^**	**0.86^∗∗∗^**	**0.88^∗∗∗^**	**−**			
SW	0.10	0.13	0.13	0.08	−		
SBW	–0.00	–0.06	–0.06	–0.04	0.18	−	
PH	–0.01	–0.04	–0.04	0.00	**0.36^∗∗∗^**	–0.16	−
TF	–0.04	–0.06	–0.06	–0.01	0.18	0.02	0.10
**(B) Diversity panel**							
FC	**0.57^∗∗∗^**	−					
LAP	**0.51^∗∗∗^**	**0.86^∗∗∗^**	−				
TC	**0.51^∗∗∗^**	**0.76^∗∗∗^**	**0.73^∗∗∗^**	−			
SW	–0.18	−**0.36^∗∗∗^**	–0.16	−**0.29^∗∗^**	−		
SBW	−**0.25^∗∗^**	−**0.43^∗∗∗^**	−**0.27^∗∗^**	−**0.29^∗∗^**	**0.40^∗∗∗^**	−	
PH	0.17	0.23^*^	0.21^*^	–0.05	0.00	−**0.29^∗∗^**	−
TF	0.02	0.12	0.12	–0.04	0.12	–0.08	0.11

TF, time to flower. Significant correlations are shown in bold. ^*^P < 0.05; ^∗∗^*P* < 0.01; ^∗∗∗^*P* < 0.001.

In the diversity panel correlations with WL tolerance were similar to the RIL population with *r* = 0.57 for flower color, leaf axil pigment (*r* = 0.51), and testa color (*r* = 0.51) again showing strong positive correlations (Table 1B). The trait stem base width exhibited a weaker correlation with WL tolerance. Analysis of testa color showed that 34 out of 110 genotypes had dark-colored testa and the rest 76 had light-colored testa (Figure 3D). The mean germination of dark testa colored genotypes was 71%, which was significantly (*P* < 0.001) higher than the mean for genotypes with light-colored testa (37%). However, the range of percent germination was similar for both dark and light testa genotypes.

### Solute Leakage/EC and WL Tolerance

**FIGURE 4:**
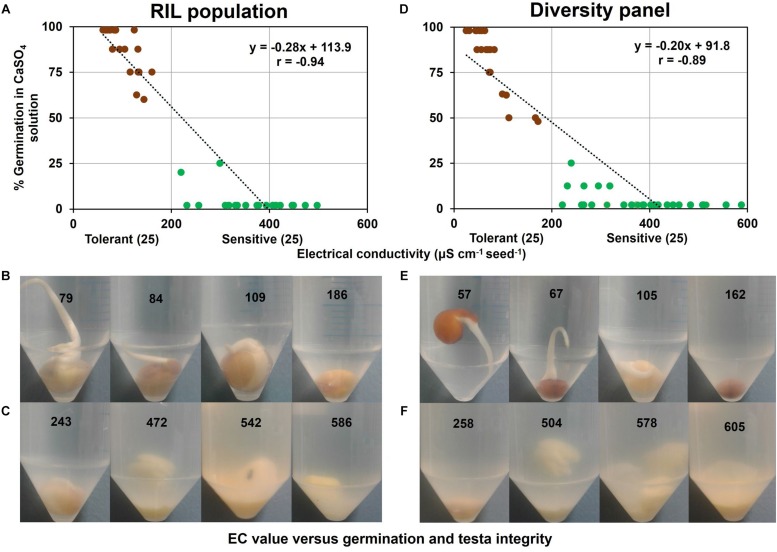
Germination versus EC of 0.5 mM CaSO_4_ solution following 6 d of seed submergence. In RIL population **(A)** association between EC and germination, **(B)** intact testa with germination in tolerant genotypes, and **(C)** dissolved testa without germination in sensitive genotypes. Similarly in diversity panel **(D)** association between EC and germination, **(E)** intact testa with germination in tolerant genotypes, and **(F)** dissolved testa without germination in sensitive genotypes. Germination was counted by observing the radical longer than 3 mm in the respective solution. The values on the centrifuge tube refer to EC values (μS cm^–1^ g^–1^ seed) measured at 6 d seed submergence. Brown and green circles in **(A)** and **(D)** refer to tolerant and sensitive genotypes, respectively.

In a sub-group of the RIL population comprising contrasting tolerant and sensitive genotypes (i.e., 25 tolerant and 25 sensitive) selected from variation in WL tolerance experiment (Figure 1), germination and EC of 0.5 mM CaSO_4_ solution following 6 d of submergence were measured and were found strongly correlated (*r* = −0.94) (Figure 4A). In this association there was a clear boundary of EC value of 200 μS cm^–1^ g^–1^ seed between tolerant and sensitive groups. All the genotypes in the tolerant group had a dark testa with a low EC (61–161 μS cm^–1^ g^–1^ seed). However, all the WL-sensitive samples, composing 22 light and 3 dark testa genotypes had higher EC (220–498 μS cm^–1^ g^–1^ seed). Visual observation showed that genotypes in the WL-tolerant group had intact testa and low EC (Figure 4B). Conversely, in the WL-sensitive group many of the genotypes showed dissolved testa and higher EC (Figure 4C).

Similarly, in a sub-group of the diversity panel (25 tolerant and 25 sensitive genotypes), germination and EC of 0.5 mM CaSO_4_ solution following 6 d of submergence were measured and were found strongly correlated (*r* = −0.89) (Figure 4D). This association again had a clear boundary of EC value of 200 μS cm^–1^ g^–1^ seed separating WL-tolerant and sensitive genotypes. In the WL-tolerant group (i.e., 20 dark and 5 light testa), all the dark testa genotypes had low EC (25–172 μS cm^–1^ g^–1^ seed) but the five light testa genotypes showed higher EC (222–374 μS cm^–1^ g^–1^ seed) as sensitive group. In contrast, all the genotypes in the WL-sensitive group (i.e., 23 light and 2 dark testa) had higher EC (240–588 μS cm^–1^ g^–1^ seed). The genotypes in the tolerant group again had visually intact testa and low EC (Figure 4E). In contrast, many of the genotypes in the sensitive group exhibited dissolved testa and high EC values (Figure 4F).

### Direct and Indirect Response to Selection for Waterlogging Tolerance

Direct and indirect responses to selection for WL tolerance were estimated from germination on waterlogged soil, as there was a strong concurrence between the germination in waterlogged soil and germination of seed submerged in CaSO_4_ solution in both RIL population (*r* = 0.95) and diversity panel (*r* = 0.95) (Supplementary Figure S1). The direct response to selection for WL tolerance was based on germination values, while the indirect responses to selection for WL tolerance were based on four secondary traits (EC, testa color, flower color, and axil pigment). All the secondary traits exhibited even higher heritability (*H*^2^ = 0.95–1.00) values than that of germination % (*H*^2^ = 0.89_RIL_; 0.87_*diversity panel*_) when grown on waterlogged soil (Table 2). Among the four secondary traits, EC had the highest genetic correlation with germination in RIL population (*r*_G_ = −1.00) and diversity panel (*r*_G_ = −0.90). Comparing the efficiency of indirect selection for germination under waterlogged conditions, among the four secondary traits EC had the highest efficiency for selection in both RIL population (CR_G_/DR_G_ = −1.10) and diversity panel (CR_G_/DR_G_ = −0.98) (Table 2).

**TABLE 2:** Heritability (*H*^2^), genetic correlation (*r*_G_) of secondary traits with germination on waterlogged soil, and the efficiency of indirect selection for germination (CR_G_/DR_G_) were estimated in the RIL population and the diversity panel.

**Traits**	**RIL population**			**Diversity panel**		
	
	***H*^2^**	***r*_G_**	**CR_G_/DR_G_**	***H*^2^**	***r*_G_**	**CR_G_/DR_G_**
Germination	0.89			0.87		
EC	0.95	−1.00	−1.10	0.98	−0.90	−0.98
Testa color	1.00	0.63	0.67	1.00	0.55	0.59
Flower color	0.99	0.67	0.72	1.00	0.61	0.66
Axil pigment	1.00	0.70	0.75	1.00	0.55	0.59

*Germination was on the basis of data from waterlogged soil. CR_*G*_ is the correlated response to indirect selection for germination based on secondary traits and DR_*G*_ is the direct response to selection for germination.*

## Discussion

Waterlogging is a major constraint to crop production globally. Genetic variation is prerequisite for any crop to mitigate WL stress, which is predicted to be more frequent and extreme with climate change in temperate-tropical cropping regions (Lobell et al., 2008). In pea variation for WL tolerance at germination has been reported for only three cultivars (Zaman et al., 2018). From these three, the present study identified the extended variation of germination/WL tolerance (5–100%) first to a RIL population from a bi-parental cross and then to a broad germplasm diversity panel. During WL, due to shortage of oxygen (Armstrong and Drew, 2002), ATP formation is inhibited (Jackson and Drew, 1984) and the oxidation–reduction state between cell membranes becomes unbalanced and membrane permeability is increased. This leads to increased solute leakage (Hsu et al., 2000) (i.e., increased EC in current experiments) and decreased germination in our study. Thus, testa integrity is an indirect evaluation of seed vigor. Furthermore, high broad-sense heritability estimates for WL tolerance at germination were found in both the RIL population (*H*^2^ = 0.89) and the diversity panel (*H*^2^ = 0.87) indicating that most of the variation observed is genetic (Visscher et al., 2008). The frequency distribution of RIL lines for germination under WL showed a continuous variation indicating polygenic control for the trait. This was reinforced by the continuous distribution for WL germination expressed in the diversity panel.

Environmental stress is a powerful force to generate local adaptation through strong directional selection and rapid evolution (Erskine, 1997; Hoffmann and Parsons, 1997). We found that the germplasm most tolerant to WL was from Africa (i.e., Ethiopia), where peas are generally sown at the start of the rains (mid-June to July) at elevations from 1800 to 3000 m a.s.l. (Telaye, 1979; Tsidu, 2012). The prevailing temperature at germination in Ethiopia is warmer than at the pea’s domestication region in the Near East where germination occurs during cool wet winter conditions. With the rains in Ethiopia being more intense than those in a Mediterranean winter, the tolerance to WL of Ethiopian genotypes is probably due to their adaptation to excess soil moisture during germination. Similar adaptive potential has been identified in lentil genotypes from Bangladesh, where the crop is often sown onto waterlogged soil in the rice-based cropping system (Malik et al., 2016; Wiraguna et al., 2017). However, such directional selection causes genetic bottleneck in plant breeding; thus, we linked WL tolerance to some phenotypic traits.

Testa color is associated with WL tolerance, for example, dark (red/black/brown) testa genotypes in wheat (Ueno and Takahashi, 1997), rapeseed (Zhang et al., 2008) and soybean (Hou and Thseng, 1991) are tolerant to WL compared to light (white/yellow) testa genotypes. The difference in tolerance between dark and light testa genotypes is probably due to the levels of phenolic compounds in the testa, as in the rapeseed study dark testa genotypes had higher levels of phenolic compounds than the sensitive light testa genotypes (Zhang et al., 2008). Higher levels of phenolic or tannin compounds in the testa are considered as a barrier to imbibition, since the dark testa genotypes of pea, faba bean (*Vicia faba* L.), and Arabidopsis (*Arabidopsis thaliana* L.) are restricted in imbibition, whereas light testa is completely permeable to water and subsequent solute leakage (Marbach and Mayer, 1974; Kantar et al., 1996; Debeaujon et al., 2000). The present study showed that dark testa genotypes both in RIL population and diversity panel had high percent of germination with lower solute leakage; in contrast, light testa genotypes had low percent of germination and higher solute leakage. Furthermore, genes of wound responsive family protein are highly upregulated in dark testa genotypes in pea during WL stress, which are involved in providing the cell with lignin and phenolic precursors to the wounded surface and provide defense to plants (Zaman et al., 2019). Therefore, it is likely that testa pigmentation plays a protective role against imbibition damage during WL stress. Additionally, in the current study, among RIL population, lines with a dark testa – similar to WL-tolerant parent Kaspa – all had pigmented leaf axils and purple/pink flowers, while the light testa lines – similar to sensitive parent BM-3 – had green un-pigmented leaf axils and white flowers. Similarly in the diversity panel, dark testa genotypes predominantly had pigmented leaf axils and purple/pink flowers, whereas the light testa genotypes had non-pigmented axils and white flowers. The exceptions were a few (6%) genotypes with dark testa and pigmented leaf axils but white flowers, indicating that the effects are not pleiotropic. Such exceptions suggest that the loci for the three traits (flower color, leaf axil pigmentation, and testa color) are linked, and thus any of the traits could be a potential marker/indicator to identify WL tolerance. This finding is consistent with genes for testa and flower color which are located in the linkage group II reported by Reid and Ross (2011). Similarly, Statham et al. (1972) found that flower color in *Pisum* is controlled by six major genes, where *A* gene is necessary for general flavonoid production in the plant, and for anthocyanin production in the flowers, axils, and pods. However, Mendel observed that colored seed coats were always associated with colored (purple) flowers, and these colored varieties possessed pigmentation in the leaf axils. By contrast, a colorless testa was always associated with white flowers and the absence of pigmentation in the leaf axils, indicating that these were pleiotropic effects of a single gene. Flower color and leaf axil pigment are reported for the first time to be associated with WL tolerance in the model crop pea in our study.

Testa integrity is a pre-requisite for germination under waterlogged stress. In the present study, testa integrity measured as EC in submerged solution showed a very strong correlation with germination for both RIL population (*r* = −0.94) and diversity panel (*r* = −0.89), indicating that testa integrity might be an effective trait for WL tolerance selection. Visual observation in the RIL population and diversity panel showed that all the genotypes in the tolerant group had intact testa with low EC; in contrast, around 90% genotypes in the sensitive group had dissolved testa with higher EC. This is consistent with sudangrass (*Sorghum sudanense* Stapf) where testa integrity is associated with germination (Hsu et al., 2000). During WL the integrity of testa is lost due to the lipid peroxidation in the testa membrane (Crawford and Braendle, 1996) by the two possible pathways – enzymatic and non-enzymatic. In the enzymatic pathway, due to decreased ATP formation during WL stress, different lipid metabolic enzymes such as lipase and lipoxygenase are induced and cause membrane damage (Rawyler et al., 1999) which is supported by the highly upregulated lipid metabolic genes in the sensitive genotype in pea during WL (Zaman et al., 2019). In the non-enzymatic pathway, excessive amount of reactive oxygen species (ROS) are accumulated during WL stress that reacts with lipids in the cell membranes cause oxidative damage and eventually cell death in the testa membrane. As a result of membrane damage in the testa, the electrolytes – in particular potassium ion (K^+^), along with seeds solutes including sugars and amino acids – are released from seeds (De Vos, 1993). Thus, we can use the amount of electrolyte leaked from the seeds as a proxy for the extent of testa leakage and tolerance under waterlogged stress. However, tolerant genotypes control the lipid peroxidation/testa integrity by neutralizing ROS activity in cells by producing increased antioxidant enzymes such as superoxide dismutase, ascorbate peroxidase, glutathione reductase, and catalase during WL (Kumutha et al., 2009). The concentrations of antioxidants are positively correlated with phenolic compounds [Canola seed, Jun et al., 2014; hazelnuts (*Corylus avellana* L.), walnuts (*Juglans nigra* L.), and pistachios (*Pistacia vera* L.), Arcan and Yemenicioğlu, 2009]; and seeds with dark testa/pigmentation exhibit higher levels of phenolic compounds, lower leakage, and higher WL tolerance than the seeds with a lighter colored testa (Zhang et al., 2008, rapeseed). In the present study, 90% dark testa genotypes showed tolerant with intact testa whereas all the light testa genotypes were sensitive with dissolved testa. Therefore, it may be inferred that the antioxidant properties of dark testa seeds are key to testa integrity/WL tolerance in pea.

Peas are a major pulse crop globally, but are more sensitive to WL than other pulses (Solaiman et al., 2007). Predictions of global warming and climate variability in South Asia suggest a change in the inter-annual precipitation pattern – alterations in the intensity of rainfall events (Sivakumar and Stefanski, 2010), and delayed monsoon rains (Li et al., 2017), which could significantly change crop productivity – particularly in pea which is highly sensitive to WL and sown as a relay crop in waterlogged soil. However, as the present study has illustrated the extent of variation in WL tolerance at germination in pea, its polygenic control and the possibilities for indirect selection for WL tolerance, the prospect is raised of adapting the pea, which originates from a rainfed Mediterranean environment, into stable production under relay-sowing in moist soils with rice. We anticipate that selection for WL tolerance at an early stage of crop growth could significantly improve the reliability of relay sowing and help climate-proof production as part of a strategy to enhance productivity in South Asia.

## Data Availability

All datasets generated for this study are included in the manuscript and/or the Supplementary Files.

## Author Contributions

MZ designed the study in consultation with AM, PK, and WE. FR was involved in the RIL development with the advancement from F_2_ to F_6_ generation. MZ conducted the experiment, analyzed the data, and wrote the first draft of the manuscript. All authors contributed to the manuscript revision and editing and approved the submitted version of the manuscript.

## Conflict of Interest Statement

The authors declare that the research was conducted in the absence of any commercial or financial relationships that could be construed as a potential conflict of interest.
