# Supplementary figures and images for: Waterlogging Tolerance at Germination in Field Pea: Variability, Genetic Control, and Indirect Selection
Source: Front Plant Sci. 2019 Jul 30;10:953. doi: 10.3389/fpls.2019.00953 (PMC6682692; doi:10.3389/fpls.2019.00953)

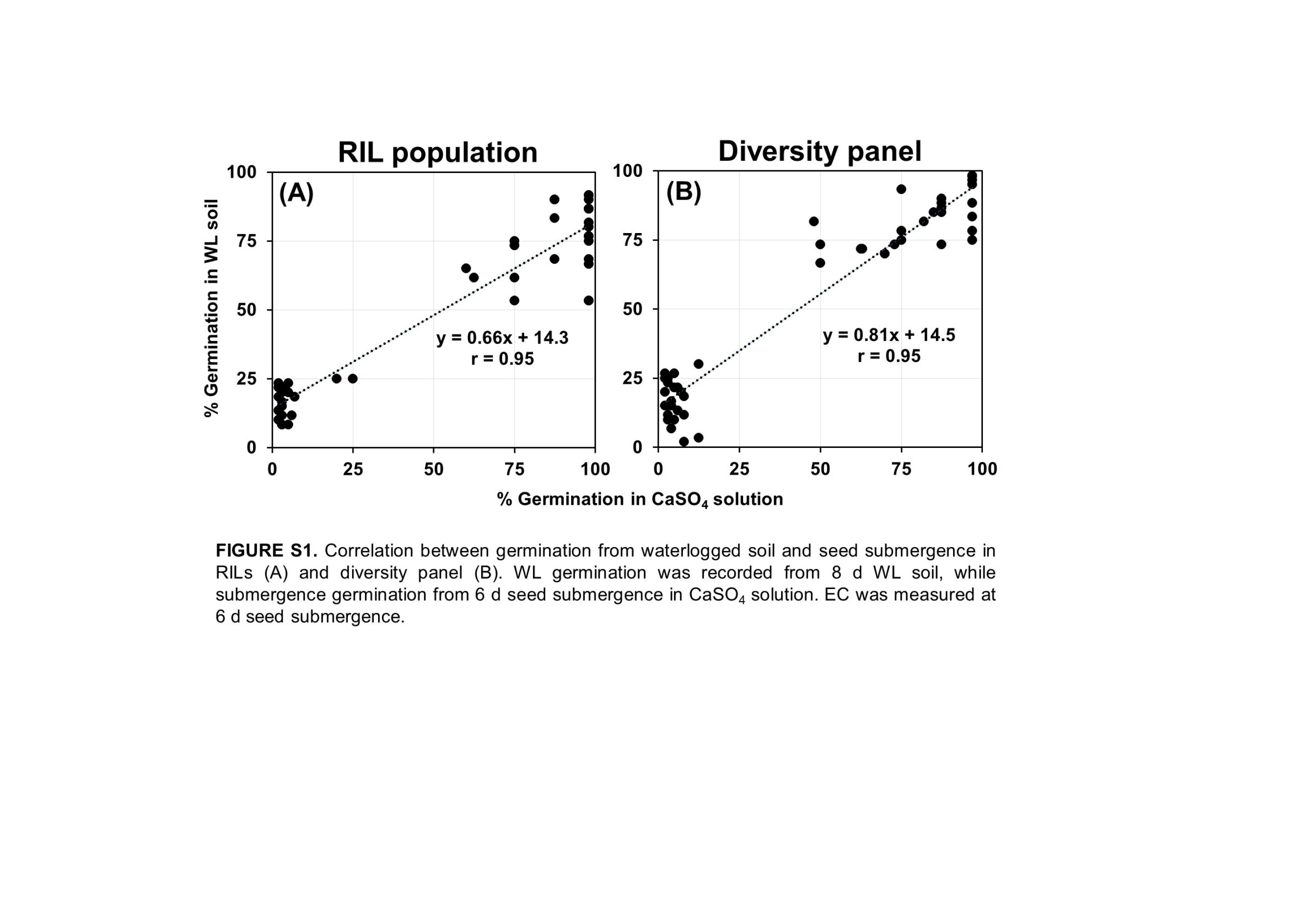

Supplement: Supplementary file 1 [file Image_1.JPEG]
